# Fabrication and Investigation of the Microwave Absorption of Nonwovens Modified by Carbon Nanotubes and Graphene Flakes

**DOI:** 10.3390/molecules28176419

**Published:** 2023-09-03

**Authors:** Wenyan Gu, Jiang Shi, Jiaqiao Zhang, Qi Jia, Chengwei Liu, Haiyan Ge, Qilong Sun, Licheng Zhu

**Affiliations:** 1 School of Textile and Clothing, Nantong University, Nantong 226019, China; gu.wy@ntu.edu.cn (W.G.); 2215320009@stmail.ntu.edu.cn (J.S.); 1915110134@stmail.ntu.edu.cn (Q.J.); 1915110158@stmail.ntu.edu.cn (C.L.); 1915110118@stmail.ntu.edu.cn (H.G.); sunqilong001@ntu.edu.cn (Q.S.); 2 School of Mechanical Engineering, Southeast University, Nanjing 211189, China; 3 National Local Joint Laboratory for Advanced Textile Processing and Clean Production, Wuhan Textile University, Wuhan 430200, China

**Keywords:** carbon nanotubes, graphene flakes, nonwovens, microwave absorption

## Abstract

This study aims to investigate the influences of carbon nanotubes (CNTs) and graphene flakes (GFs) on the microwave absorption performance of nonwovens. Nonwovens were modified with CNTs and GFs through an impregnation method, creating a series of absorption samples with different carbon nanomaterial contents. Then the absorption performance of the samples was tested on both sides in the X-band (8.2~12.4 GHz) and the Ku-band (12~18 GHz) using the arch method. The experimental results showed that the absorption performance of GF-impregnated nonwovens was superior to that of CNT-impregnated nonwovens, and the overall absorption performance in the Ku-band was better than in the X-band. At a CNT content of 5 wt.%, the reflection loss of the impregnated nonwovens on the backside reached a minimum of −14.06 dB and remained below −10 dB in the 17.42~17.88 GHz frequency range. The sample fabricated with 4 wt.% GFs in the impregnation solution exhibited the best absorption performance, with minimum reflection losses of −15.33 dB and −33.18 GHz in the X-band and Ku-band, respectively. When the GFs were at 3 wt.%, the absorption bandwidth below −10 dB reached 4.16 GHz. In contrast to CNT-impregnated nonwovens, the frontside of GF-impregnated nonwovens demonstrated better absorption performance in the Ku-band. The results of this work provide experimental data support for the fabrication and application of microwave absorption materials.

## 1. Introduction

The demand for electromagnetic wave absorption materials has been steadily increasing due to their wide range of applications in various fields, such as radar systems, wireless communication, and electromagnetic interference shielding [1,2,3]. However, electromagnetic radiation can have negative impacts on the surrounding environment and ecological systems [4,5]. Prolonged exposure to high-intensity electromagnetic fields may adversely affect human health [6]. Electromagnetic radiation can cause both ionizing and non-ionizing effects, damaging human cells and tissues [7,8], thereby increasing the risk of diseases such as cancer, reproductive issues, and immune system abnormalities. Microwaves are a wavelength range within the electromagnetic spectrum, and microwave absorption plays a crucial role in mitigating electromagnetic wave hazards. Among various materials, nonwovens have drawn considerable attention as potential candidates for microwave absorption due to their light weight, flexibility, and ease of processing [9,10,11].

In response to the serious threat of electromagnetic pollution, the study of absorbing materials based on nonwovens holds significant importance [12]. Absorbing materials can effectively absorb the energy of microwaves, converting them into weak thermal energy instead of reflecting and propagating [13,14]. By absorbing electromagnetic radiation within the material, the propagation speed of microwaves can be reduced, and their reflection can be minimized, thereby lowering the range and intensity of radiation. The absorption performance is typically evaluated using reflection loss [15,16]. Reflection loss refers to the degree to which a material reflects electromagnetic waves at a specific frequency and is expressed as a negative value. A higher reflection loss value indicates a lower reflection of electromagnetic waves and a stronger ability to absorb them, thus indicating better absorption performance. A reflection loss value below −10 dB indicates that the material can effectively absorb electromagnetic waves over most frequency ranges, making it an ideal absorption effect.

Currently, there is a wide variety of common microwave-absorbing nanomaterials. Similarly, the nanostructures are diverse, from zero-dimensional (0D), such as nanosphere; one-dimensional (1D) nanowire; and two-dimensional (2D) nanosheet to complex three-dimensional (3D) spatial structures [17]. MXene is a novel type of two-dimensional material composed of transition metal carbide or nitride layers. Due to their unique structure and properties, MXene materials have garnered significant attention in the field of microwave absorption [18]. MXenes have been explored as electromagnetic shields in the place of highly conductive yet dense and corrosion-susceptible metals, but individual MXene materials do not meet the prerequisites for real application [19]. Furthermore, MXene is expensive, and its production process is not yet mature enough to be applied in wearable microwave-absorbing nonwovens.

Carbonaceous materials are considered promising for efficient microwave absorption due to their synergistic loss mechanism as well as their tunable architecture design [20]. Carbon nanotubes (CNTs) and graphene flakes (GFs) are carbon nanomaterials with exceptional electrical and mechanical properties [21,22,23], making them promising candidates for enhancing the absorption performance of nonwovens [24,25,26]. The incorporation of these nanomaterials into the fabric matrix can significantly modify their electromagnetic properties and improve their ability to absorb and attenuate microwaves. In recent research, Xiaojun Chen et al. [27] revealed that CNTs, owing to their high dielectric constant, remarkable thermodynamic stability, and low density, exhibit promising microwave absorption performance when compounded with various matrix materials. In recent years, metals such as Fe [28], Co [29], Ni [30], and Ag [31] have often been used in combination with CNTs to enhance their permeability and interface interactions, thereby improving microwave absorption performance. However, research on the microwave absorption capacity of nonwovens modified with pure carbon nanotubes as absorbers has been overlooked. Fanbin Meng et al. [32] suggest that graphene flakes, due to their low density, high specific surface area, strong dielectric loss, and high electronic conductivity, have emerged as microwave absorption materials for high-performance electromagnetic wave attenuation. Similarly, incorporating other lossy materials to enhance the conductivity and limited loss of graphene materials has been extensively studied [33,34]. However, research on the microwave absorption performance of nonwovens modified solely with graphene is lacking. Investigating the absorption performance of nonwovens modified with CNTs and GFs can serve as a benchmark for further studies.

In this study, we impregnated polyester needle-punched nonwovens with polyurethane (PU) solutions containing different concentrations of CNT and GF particles. The objective was to investigate the impact of these nanomaterials on the absorption performance of the nonwovens in the X-band and Ku-band frequency ranges. By analyzing the reflection loss data obtained from both the front and backsides of the impregnated nonwovens, we aimed to identify the optimal composition and configuration for achieving enhanced absorption performance. The results of this research contribute to the development of advanced microwave absorption materials and provide valuable insights for their practical applications in the field of microwave management.

## 2. Experimental Results and Discussion

### 2.1. Microscopic Morphology Characterization

**Figure 1:**
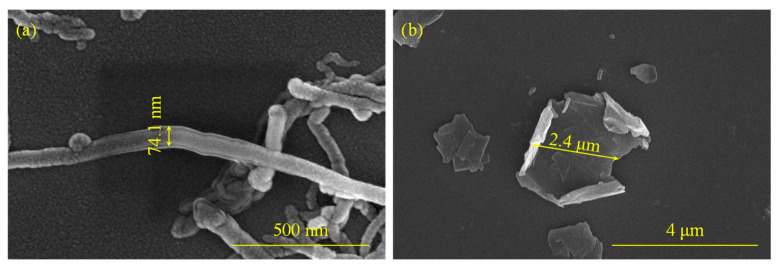
Microscopic morphology and dimensions of nanomaterials: (**a**) CNT; (**b**) GF.

**Figure 2:**
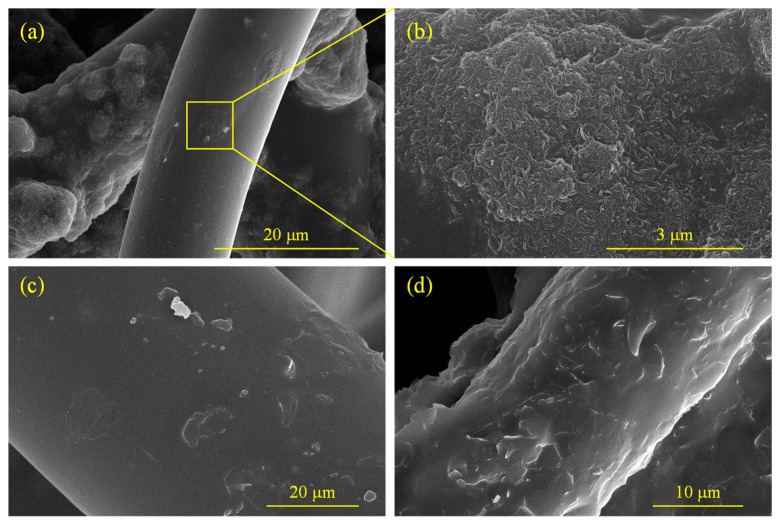
Distribution of nanomaterials on the fiber surface: (**a**) C-6; (**b**) magnified view; (**c**) G-2; (**d**) G-5.

The details of the morphology of the CNTs and GFs in the experiment are illustrated in Figure 1a,b respectively. It can be observed that the diameter of the CNT was 74.1 nm, while the size of the GF was 2.4 μm. Figure 2a,b show the CNTs adhering to fibers. CNTs were wrapped with PU and tightly adhered to the fiber surface, and some agglomerations of CNTs still existed. Figure 2c,d display the GFs adhering to the fiber surface. As the content of GFs increased from 2 wt.% to 5 wt.%, the quantity of GFs on the fiber surface noticeably rose.

**Figure 3:**
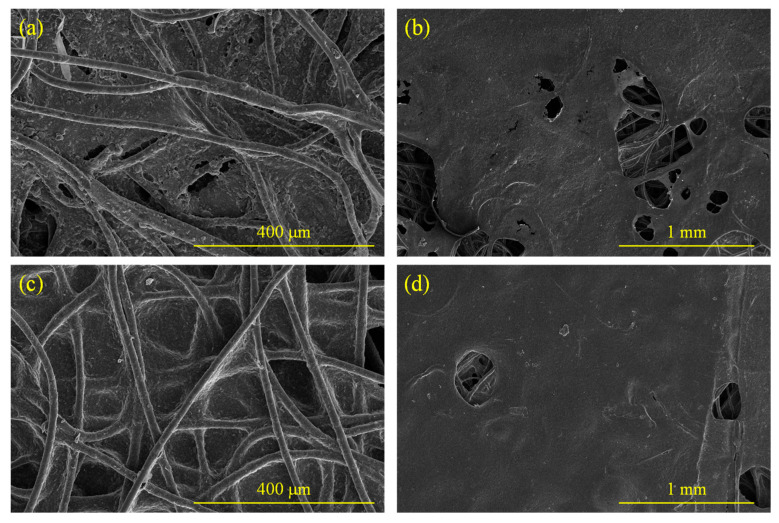
Surface images of the samples: (**a**) frontside of C-5; (**b**) backside of C-5; (**c**) frontside of G-5; (**d**) backside of G-5.

Figure 3a–d, respectively, present the microscopic images of the front and backsides of samples C-5 and G-5. It can be observed that the front and backsides of the materials exhibited distinct characteristics. On the frontsides, the shape of fibers similar to nonwoven materials was relatively clear, and the pores generated by fiber interweaving were randomly distributed, while on the backsides, the characteristics of overall casting were shown, with slight fiber traces faintly discernible, and the entire material surface showed the dispersion of small particles.

**Figure 4:**
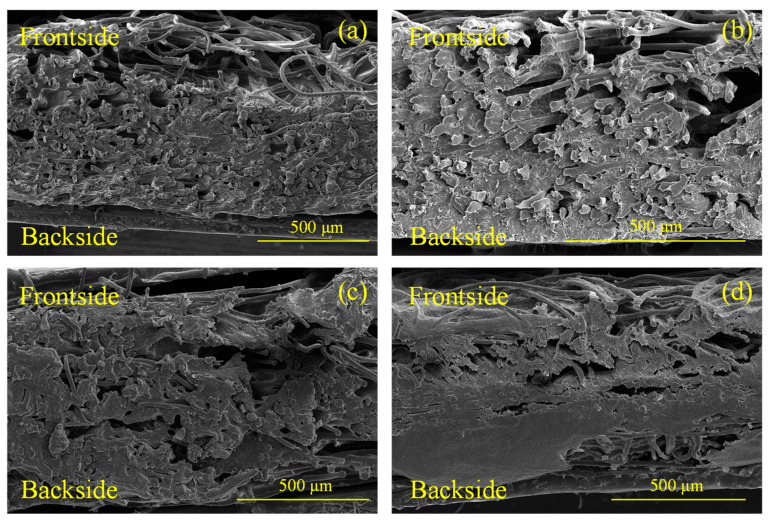
Cross-sectional images: (**a**) C-2; (**b**) C-6; (**c**) G-2; (**d**) G-6.

**Figure 5:**
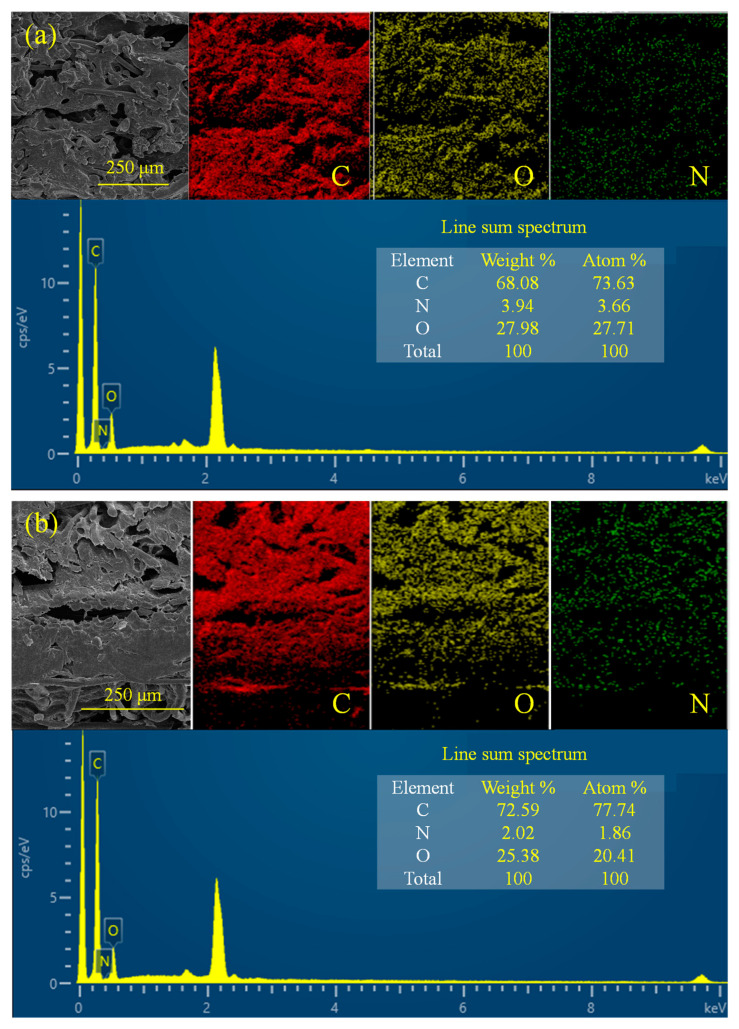
Energy spectra: (**a**) G-2; (**b**) G-6.

Figure 4a–d present the cross-sectional images of samples C-2, C-6, G-2, and G-6, respectively. It can be observed that the distribution of PU gradually decreased from the backsides of the samples to the frontsides. The cross-sections of the samples exhibited a gradient distribution, with the structure being denser on the backside and more porous on the frontside. Figure 5a,b show the energy spectra of the interfaces of G-2 and G-6. It can be seen that the specimens were primarily composed of three elements: carbon (C), oxygen (O), and nitrogen (N). In both spectra, C content was the highest, while N content was the lowest. As the content of GFs increased from 2 wt.% to 6 wt.%, the C element content also increased from 68.08 wt.% to 72.59 wt.%.

### 2.2. Influences of CNTs on the Absorption Performance of Nonwovens

#### 2.2.1. Absorption Performance of CNT-Impregnated Nonwovens in X-Band

**Figure 6:**
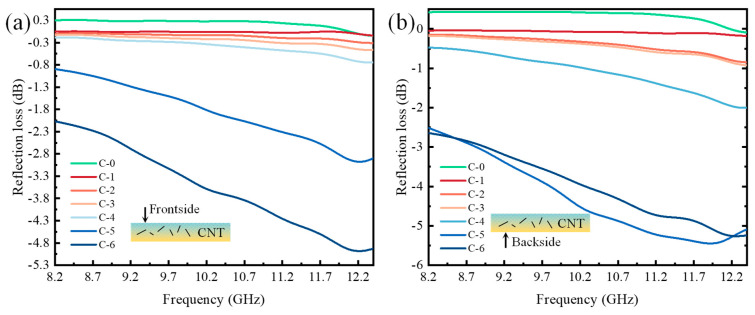
The reflection losses of CNT-impregnated nonwovens in X-band: (**a**) frontside (**b**) backside.

The curves of reflection loss measured on the frontside of the samples within the X-band (8.2~12.4 GHz) are shown in Figure 6a. It can be observed that for C-0 and groups C-1 to C-4, the reflection loss fluctuated insignificantly. However, groups C-5 and C-6 exhibited a noticeable decreasing-then-increasing trend. The minimum reflection loss showed a decreasing trend as the CNT content increased. Therefore, it can be concluded that the absorption performance on the frontside of the impregnated needle-punched nonwovens gradually improved with the increase in CNT content. When the CNT content was 6 wt.%, the minimum reflection loss on the frontside of the impregnated nonwovens was reached at 12.21 GHz, which is −4.93 dB. At this point, there was no bandwidth below −10 dB within the X-band.

In the X-band, the data obtained from the backside testing of the samples are shown in Figure 6b. Similar to the results from the frontside testing, the reflection losses of C-0 and groups C-1 to C-3 fluctuated insignificantly. However, groups C-4 to C-6 exhibited a noticeable decreasing-then-increasing trend, with C-5 demonstrating better absorption performance than C-6. Therefore, with the increase in CNT content, the absorption performance on the backside of CNT-impregnated needle-punched nonwovens first increased and then decreased. When the CNT content was 5 wt.%, the minimum reflection loss on the backside of the impregnated nonwovens was achieved at 11.92 GHz, which was −5.45 dB. At this point, there was still no bandwidth below −10 dB within the X-band.

#### 2.2.2. Absorption Performance of CNT-Impregnated Nonwovens in Ku-Band

**Figure 7:**
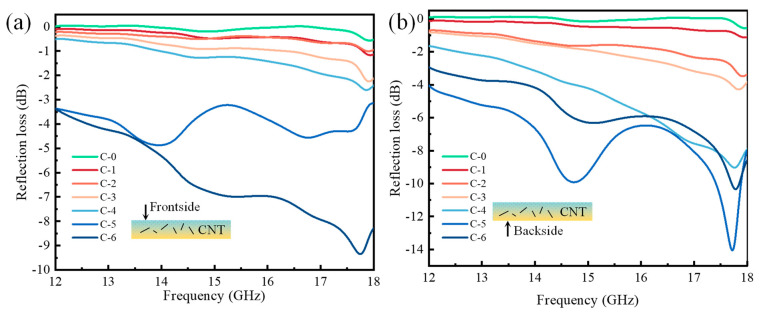
The reflection losses of CNT-impregnated nonwovens in Ku-band: (**a**) frontside (**b**) backside.

In the Ku-band frequency range, the data obtained from frontside testing of the samples are shown in Figure 7a. The reflection losses of C-0 and groups C-1 to C-4 still fluctuated within a relatively small range and showed an overall decreasing trend. Group C-5 exhibited a curve variation in reflection loss, but the values remained in the range of −5~−3 dB, making it difficult to further reduce the reflection loss. Group C-6 showed a significant decreasing-then-increasing trend in reflection loss, and its absorption performance was better than the other groups. Therefore, in the Ku-band, the reflection loss on the frontside of the impregnated nonwovens decreased with an increase in CNT content, leading to an enhancement in absorption performance. When the CNT content was 6 wt.%, the reflection loss reached −9.35 dB at 17.75 GHz. However, within the Ku-band, there was no bandwidth below −10 dB.

In the Ku-band, the data obtained from backside testing of the samples are shown in Figure 7b. The reflection losses of the groups C-0 and C-1 exhibited similar results, with relatively small fluctuations. Groups C-2 and C-3 started to show a decreasing-then-increasing trend in reflection loss. Groups C-4 to C-6 exhibited larger fluctuations in reflection loss, and the range of fluctuations also increased. Among them, both the C-5 and C-6 groups not only surpassed the minimum reflection loss peak but also showed two frequency bands with reflection losses below −10 dB. Overall, the absorption performance of C-5 was better than that of C-6. Therefore, within the frequency range of 12~18 GHz, the minimum reflection loss of CNT-impregnated nonwovens exhibited a decreasing-then-increasing trend with an increase in CNT content, indicating that the absorption performance increased and then decreased. When the CNT content was 5 wt.%, the reflection loss on the backside of the impregnated nonwoven reached −14.06 dB at 17.72 GHz, and the frequency band below −10 dB was from 17.42 GHz to17.88 GHz. When the CNT content was 6 wt.%, the reflection loss on the backside of the impregnated nonwoven reached −10.34 dB at 17.78 GHz, and the frequency band below −10 dB was from 17.72 GHz to 17.85 GHz.

### 2.3. Influences of GFs on the Absorption Performance of Nonwovens

#### 2.3.1. Absorption Performance of GF-Impregnated Nonwovens in X-Band

**Figure 8:**
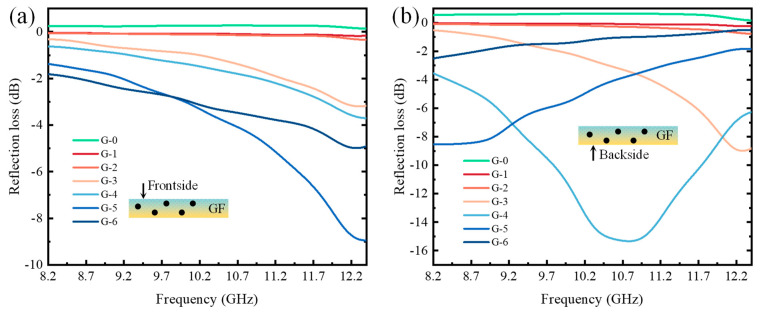
The reflection losses of GF-impregnated nonwovens in X-band: (**a**) frontside (**b**) backside.

In the X-band frequency range, the data obtained from frontside reflection loss testing of the samples are shown in Figure 8a. With an increase in GF content, the reflection loss of each group of samples generally decreased, indicating that the absorption performance of the samples was affected by the GF content. Observing groups G-0, G-1, and G-2, it can be seen that with lower GF content, the reflection losses of the samples fluctuated less, suggesting that a small quantity of graphene flakes did contribute to absorption, but the absorption performance was relatively poor. As the GF content increased, the reflection loss curves of the samples showed significant fluctuations, indicating that the absorption effect of GFs was greatly enhanced. In addition, the minimum reflection loss exhibited a decreasing-then-increasing trend, indicating that the absorption performance of the samples fluctuated, with an initial increase and then a decrease. When the GF content was 5 wt. %, the frontside reflection loss of the samples reached −8.95 dB at 12.38 GHz. Although there was no frequency band below −10 dB, it still demonstrated a fairly great absorption performance. Therefore, in the frequency range of 8.2~12.4 GHz, the GF-impregnated samples exhibited great absorption performance on the frontside, and they particularly performed well in the mid-to-high frequency range.

The data obtained from backside reflection loss testing of the samples in the frequency range of 8.2~12.4 GHz are shown in Figure 8b. Similar to the frontside reflection loss, the reflection loss curves of the G-0, G-1, and G-2 groups mostly exhibited straight lines, with slight reductions in loss values, but their fluctuation ranges remained limited, indicating less-than-ideal absorption performance. However, for the G-5 and G-6 groups, their reflection loss curves showed a gradual increase in the X-band. This is believed to be because their peaks appeared in the frequency range before 8.2 GHz, resulting in this trend in the X-band. Compared to the frontside reflection loss curves, the G-3 curve showed little difference other than a slight reduction in loss values. On the other hand, the G-4 curve exhibited significant changes, showing a sharp decrease followed by an increase in the X-band and a frequency range with reflection loss below −10 dB, indicating excellent absorption performance when the GF content was 4 wt.%. At 10.78 GHz, the backside reflection loss of the sample reached −15.33 dB, with a frequency range below −10 dB from 9.79 GHz to 11.74 GHz.

Comparing Figure 8a with Figure 8b, it can be found that the backside absorption performances of G-1, G-2, G-3, and G-4 were better than those of the frontside in the X-band, while the frontside absorption performances of G-5 and G-6 were better than those of the backside. With the increase in GF content in the impregnation solution, a slight decrease in flowability and a slightly accelerated curing speed happened in the GF-modified impregnation solution. Thus, a slight pressure had to be employed to slowly blade coat the GF-modified impregnation solution into the nonwoven material to ensure that the entirety of the nonwoven material could be totally soaked by the GF-modified impregnation solution until the solution was set. Owing to the high weight and viscosity of polyurethane, blade coating under a slight pressure inevitably caused the GF-modified impregnation solution, which was originally at the bottom of the mold and adhered to the backside of the nonwovens, to be squeezed up to the frontside of the nonwovens. After the external force disappeared, the impregnation solution redistributed among the fiber gaps with the actions of both gravity and the capillary effect. However, besides the adsorption activity of GFs, the weakened fluidity and shortened curing time of the GF impregnation solution caused a certain amount of GF-modified impregnation solution to be left both on the frontside and along the thickness of the nonwovens. When the amount of graphene was low, such as in G1, G2, G3, G4, the enhancement of reflection loss barely happened, with only a little GF redistribution along the thickness and frontside of the absorbing nonwovens. However, when the amount of graphene was high, such as in G-5 and G-6, due to the increase in the quantity of GFs on the frontside and along the thickness, the reflection losses on the frontside were significantly better than those of the backside.

#### 2.3.2. Absorption Performance of GF-Impregnated Nonwovens in Ku-Band

**Figure 9:**
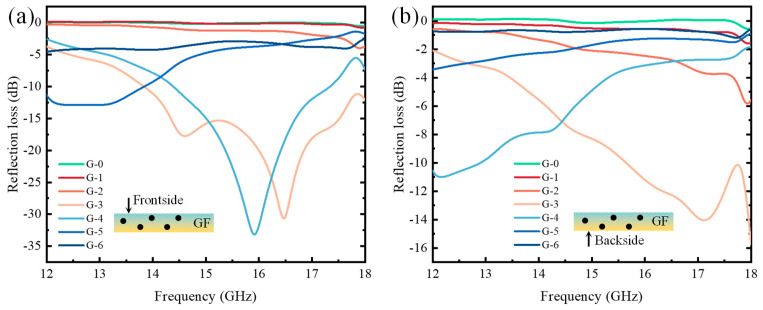
The frontside and backside reflection losses of GF-impregnated nonwovens in Ku-band: (**a**) frontside (**b**) backside.

The frontside reflection loss test results of the samples in the Ku-band are shown in Figure 9a. Among them, the reflection loss fluctuations of the G-0, G-1, G-2, and G-6 groups were reduced, and their reflection loss values were all greater than −5 dB. The G-3 and G-4 groups both showed distinct reflection loss peaks, indicating good absorption performance. Additionally, the G-5 group also exhibited a frequency band with reflection loss below −10 dB.

When the GF content was 3 wt.%, the frontside reflection loss of the sample reached −30.67 dB at 16.47 GHz, and the frequency band with reflection loss below −10 dB was 13.84~18.00 GHz. When the GF content was 4 wt.%, the minimum frontside reflection loss of the sample was −33.18 dB at 15.93 GHz, and the frequency band with reflection loss below −10 dB was 14.93~17.34 GHz. When the GF content was 5 wt.%, the frontside reflection loss of the sample reached −12.90 dB at 12.94 GHz, and the frequency band with reflection loss below −10 dB was 12.00~13.84 GHz.

Therefore, in the Ku-band frequency range, the frontside absorption performance of the samples was generally excellent, with extremely low reflection loss values of −30.67 dB and −33.18 dB and three frequency bands with reflection losses below −10 dB. This indicates that the nonwoven samples impregnated with GFs exhibited outstanding absorption performance.

The data obtained from the backside reflection loss tests in the 12~18 GHz frequency range are shown in Figure 9b. Overall, the curves of G-0, G-1, and G-6 exhibited relatively small fluctuations, and even the reflection losses of the G-2 and G-5 samples showed no significant variation, with peak values exceeding −10 dB, while the reflection curves of the G-3 and G-4 groups displayed opposite trends. They did not exhibit a significant influence on the reflection and absorption of microwaves, indicating good absorptive properties. In the Ku-band, when the GF content was 3 wt.%, the backside reflection loss of the samples reached −15.74 dB at 18.00 GHz, with a frequency band below −10 dB from 15.65 GHz to 18.00 GHz. When the GF content was 4 wt.%, the backside reflection loss of the samples was −11.01 dB at 12.17 GHz, with a frequency band below −10 dB from 12.00 GHz to 12.90 GHz. Apart from the outstanding reflection losses of the G-3 and G-4 samples, the reflection loss values of the other samples were all greater than −10 dB. By comparing Figure 9a with Figure 9b, it can be observed that in the Ku-band, the frontside reflection loss of GF-impregnated nonwovens was better than that of the backside. The samples in Figure 9a,b were the same as the samples in Figure 8a,b; thus, the reasons considered in this part are still consistent with the aforementioned. The difference is that due to the increase in the absorption frequency band, the absorption thickness decreased. GF-integrated nonwovens can precisely provide the required effective absorption position along the direction of the thickness, so they exhibit better reflection loss on the frontside than on the backside.

### 2.4. Discussion

For needle-punched nonwovens, a reflection loss smaller than −10 dB indicates effective absorption of microwaves over most of the frequency ranges, which is considered to be an ideal absorptive performance. The results of reflection losses below −10 dB are summarized in Table 1. It can be observed that compared to the X-band, the modified impregnated nonwovens exhibited better absorption performance in the Ku-band. The absorption performance of GF-impregnated nonwovens was superior to CNT-impregnated nonwovens, and the backside absorption performance was generally better than the frontside absorption performance.

In the frequency range of 8.2~12.4 GHz, with the increase in CNT content, the absorption performance of the needle-punched nonwoven substrate gradually improved, and the minimum reflection loss decreased. The C-5 group exhibited the best absorption performance, reaching a minimum reflection loss of −5.45 dB.

The overall absorption performance of CNT-impregnated nonwovens in the X-band was relatively poor. In the Ku-band, the absorption performance of CNT-impregnated nonwovens improved with the increasing content of CNTs. When the CNT content was 5 wt.%, the backside reflection loss of the impregnated nonwoven reached a minimum value of −14.06 dB. The C-5 and C-6 groups showed excellent absorption performance, with the backside reflection losses being below −10 dB in the frequency ranges of 17.42~17.88 GHz and 17.72~17.85 GHz, respectively.

The absorption performance of GF-impregnated nonwovens shows a trend of first increasing and then decreasing with the increase in GF content. The absorption performance was most significant when the impregnation solution contained 3 wt.% or 4 wt.% GFs. In the X-band, the nonwoven with 4 wt.% GF content exhibited the best absorption performance on the backside, achieving a minimum reflection loss of −15.33 dB. In this case, the absorption band below −10 dB was in the frequency range of 9.79~11.74 GHz. In the Ku-band, the frontside of the nonwoven with 4 wt.% GF content demonstrated a minimum reflection loss of −33.18 dB. On the other hand, the frontside of the nonwoven with 3 wt.% GF content had the widest effective absorption bandwidth, which spanned 4.16 GHz. Unlike the CNT-impregnated nonwovens, the frontside of the GF-impregnated nonwovens showed better absorption performance than the backside in the Ku-band. This difference is preliminarily attributed to the distinct morphology and dielectric properties of CNTs and GFs. The size of GFs is much larger than that of CNTs, and they have a higher specific surface area. A large number of GF nanoparticles were dispersed between the nonwovens, generating tortuous paths. The nonwovens impregnated with GFs had a gradient structure, with a dense backside and a loose frontside. After the microwave entered the modified nonwovens from the frontside, the microwave paths were blocked by GFs, resulting in multiple reflection losses. This resulted in a better absorbing effect in the frontside of GF-modified nonwovens than in the backside.

## 3. Materials and Methods

### 3.1. Raw Materials

Water-based polyurethane (YC-601C, concentration 43%) and thickening agent (YC-100B) were provided by Yuanchen New Materials Technology Co., Ltd. (Hefei, China). Graphene flake solution (concentration: 18%) and carbon nanotube solution (concentration: 15%) were purchased from Nakate New Materials Technology Co., Ltd. (Suqian, China). Both of them were dispersed in water. Dispersant (6508) and defoamer (DF904A) were sourced from Aohe New Materials Co., Ltd. (Guangzhou, China). Needle-punched polyester nonwovens that were 1 mm thick were provided by Yiwu Short Flute E-commerce Co., Ltd. (Yiwu, China). Purified water was self-made in the laboratory.

### 3.2. Sample Preparation

To investigate the influence of the addition of CNTs and GFs on the microwave absorption performance of nonwovens, polyurethane was mixed with carbon nanotube solution and graphene flake solution, respectively, according to Table 2 to obtain fourteen kinds of PU solutions, of which seven had certain CNT mass ratios and seven had certain GF mass ratios.

**Figure 10:**
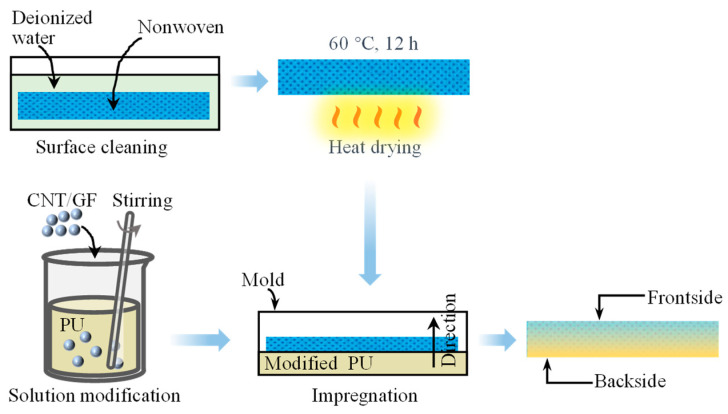
Preparation process of the samples.

In the current work, these modified PU solutions and nonwoven materials were processed into impregnated nonwoven samples, as shown in Figure 10. Firstly, a piece of pre-cut nonwoven substrate was washed with deionized water to remove impurities and fragments from material, ensuring the cleanliness of the samples. Next, the substrate was placed in an electrically heated oven and dried at a constant temperature of 60 °C for 12 h, followed by a natural cooling of 12 h. Then a kind of modified PU solution was poured into a 300 × 300 mm^2^ mold, ensuring that the solution fully covered the bottom surface of the mold. Subsequently, a piece of nonwoven material was placed into the mold, the edges were adjusted with tweezers, and it was pressed to ensure a complete impregnation. After that, the sample was naturally dried for 48 h. Finally, the sample was removed from the mold.

Fabrication of GF-impregnated nonwovens followed a similar process to CNT-impregnated nonwovens. The difference was that, with the increase in the quantity of GFs, the fluidity of the GF-modified PU solution was slightly weakened and the curing time was slightly reduced. Thus, a slight pressure was employed to carry out a slow blade-coating operation during the GF impregnation to ensure that the GF-modified impregnation solution soaked the whole sample before the solution set.

It is noted that the impregnation was conducted on one side only. The side that came into contact with the impregnation solution was defined as the backside and the other side as the frontside. During the experiment, the absorption performances of both sides were tested separately.

### 3.3. Measurement and Characterization

**Figure 11:**
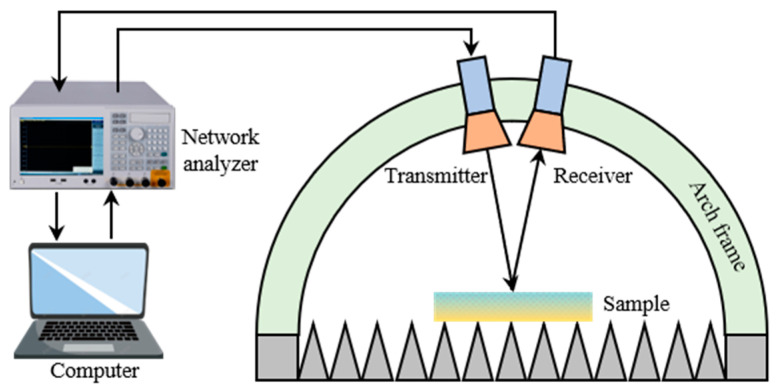
The structure of the arch method testing system.

In this study, the arch method was adopted to test the microwave absorption performance of samples, and the structure of the arch method testing system is illustrated in Figure 11 [35]. The test system operates within the frequency range of X-band and Ku-band, with a requirement that the upper and lower limits of the tested samples have a deviation of no more than 1 mm. The testing system uses a scanning method, with requirements for a measurement dynamic range greater than 40 dB and a positioning alignment error of the sample board of less than 0.05°. The experimental environment was controlled within a temperature range of 20~26 °C and a relative humidity below 80%.

The reflection loss testing steps were as follows. Step 1: the network analyzer (AV3672C type, China Electronics Technology Group Corporation, Beijing, China) was started for preheating; step 2: the parameters were input as prompted by the program on the computer to calibrate the testing system; step 3: the reflection loss of the standard sample was measured; step 4: the prepared samples were tested on both frontsides and backsides; step 5: data processing was performed and graphical representations were generated.

For the observation of the micro-surface morphology of the samples, a scanning electron microscope (SEM) (EVO15 model, Carl Zeiss manufacturer, Oberkochen, Germany) was used.

## 4. Conclusions

In the current work, 1 mm thick polyester needle-punched nonwovens were impregnated with PU-modified impregnation solutions containing different concentrations of CNT and GF particles. The main conclusions are as follows:

(1) The absorption performance of the GF-impregnated nonwovens was found to be superior to that of the CNT-impregnated nonwovens, and the samples showed overall better absorption performance in the Ku-band compared to the X-band.

(2) When the CNT content was 5 wt.%, the minimum reflection loss on the backside of the impregnated nonwoven reached −14.06 dB, and the reflection loss was below −10 dB in the frequency range of 17.42~17.88 GHz.

(3) For the nonwovens impregnated by a 4 wt.% of GFs, they exhibited the best absorption performance with minimum reflection losses of −15.33 dB and −33.18 dB in the X-band and Ku-band, respectively. Moreover, when the GF content was 3 wt.%, the absorption bandwidth below −10 dB reached 4.16 GHz.

(4) Unlike the CNT-impregnated nonwovens, the frontside of the GF-impregnated nonwovens showed better absorption performance in the Ku-band compared to the backside.

Therefore, loading CNT and GF particles on needle-punched nonwovens can effectively enhance the absorption performance of the product.

At present, we are only conducting single-factor experiments with the main purpose of clarifying the impact mechanisms of different types of nanomaterials on the microwave absorption performance of nonwovens. Next, we will conduct a multi-factor experiment to investigate the effects of a CNT and GF mixture on the absorption performance of nonwovens.

## Figures and Tables

**Table 1:** Summary of reflection loss results below −10 dB.

Particles	Direction	No.	Minimum RL/dB	Frequency/GHz	Bandwidth < −10 dB/GHz	Band Type
CNTs	Backside	C-5	−14.06	17.72	17.42~17.88	Ku-band
	Backside	C-6	−10.34	17.78	17.72~17.85	Ku-band
GFs	Backside	G-4	−15.33	10.78	9.79~11.74	X-band
	Frontside	G-3	−30.67	16.47	13.84~18.00	Ku-band
	Frontside	G-4	−33.18	15.93	14.93~17.34	Ku-band
	Frontside	G-5	−12.90	12.94	12.00~13.84	Ku-band
	Backside	G-3	−15.74	18.00	15.65~18.00	Ku-band
	Backside	G-4	−11.01	12.17	12.00~12.90	Ku-band

**Table 2:** Experimental parameters.

No.	CNT Content/wt.%	No.	GF Content/wt.%
C-0	0	G-0	0
C-1	1	G-1	1
C-2	2	G-2	2
C-3	3	G-3	3
C-4	4	G-4	4
C-5	5	G-5	5
C-6	6	G-6	6

## Data Availability

Not applicable.
